# Development and optimization of PFAS extraction in soil’s headspace followed by multidimensional gas chromatography and mass spectrometry

**DOI:** 10.1007/s00216-025-06266-4

**Published:** 2025-12-16

**Authors:** Maria Chiara Corviseri, Allan Dos Santos Polidoro, Claudia Stevanin, Luisa Pasti, Flavio Antonio Franchina

**Affiliations:** 1 Department of Environmental and Prevention Sciences, University of Ferrara, Via L. Borsari 46, 44121 Ferrara, Italy; 2 Department of Chemical, Pharmaceutical, and Agricultural Sciences, University of Ferrara, Via L. Borsari 46, 44121 Ferrara, Italy

**Keywords:** Non-targeted analysis, Fluorinated compounds, Dynamic headspace, Comprehensive two-dimensional gas chromatography, Persistent organic pollutants

## Abstract

Per- and polyfluoroalkyl substances (PFAS) are a large class of synthetic “forever chemicals” due to their exceptional stability resulting from the strong carbon-fluorine bond, which makes them persistent, bioaccumulative, and associated with health and ecological risks. Despite their relatively high molecular weights, many PFAS exhibit sufficient volatility to be analyzed by gas chromatography coupled with mass spectrometry (GC-MS). In this study, a novel analytical workflow was developed for extracting and analyzing (semi-)volatile PFAS in soil samples, combining dynamic headspace extraction (DHS) using thermal desorption (TD) tubes with both one-dimensional gas chromatography (1D-GC) and comprehensive two-dimensional gas chromatography (GC×GC), each coupled with time-of-flight mass spectrometry (TOFMS). Nine target PFAS compounds were selected, comprising fluorotelomer alcohols (FTOHs), fluorotelomer acrylates (FTAc), and sulfonamide-based derivatives (N-MeFOSA, N-EtFOSA, N-MeFOSE, and N-EtFOSE). Building on a prior aqueous-sample study, Tenax TA was identified as the optimal sorbent based on recovery and reproducibility. Extraction volumes of 1 L, 2 L, and 5 L were evaluated, with 1 L providing the best recovery-reproducibility balance. Three different conditions (no solvent, pure water, and 1:1 (*v/v*) water-methanol mixture) were tested to evaluate solvent effects. The water-methanol mixture yielded the highest recovery, ensuring consistent reproducibility. Detection limits as low as 7.02 pg g⁻^1^ were achieved, demonstrating excellent sensitivity. In a soil case study, the use of GC×GC-TOFMS enabled the identification of 106 additional environmentally relevant compounds. Overall, the DHS-TD-GC×GC-TOFMS approach offers a sensitive, robust platform for quantifying (semi-)volatile PFAS and screening other trace contaminants, supporting comprehensive environmental monitoring.

**Graphical Abstract:**

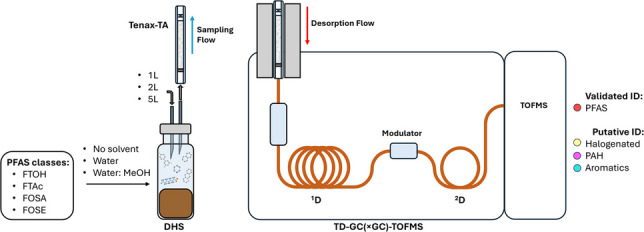

**Supplementary Information:**

The online version contains supplementary material available at 10.1007/s00216-025-06266-4.

## Introduction

Per- and polyfluoroalkyl substances (PFAS) comprise a vast family of anthropogenic chemicals that contain at least one perfluorocarbon moiety (–C_n_F_2n_–) [1]. Chemically, PFAS can be described as amphiphilic molecules, since they consist of a hydrophobic carbon chain, in which the hydrogen atoms are partially (poly-) or fully (per-) substituted with fluorine atoms, attached to a “head” composed of a polar functional group such as carboxylic acids, sulfonic acids, or alcohols [2]. Furthermore, the low polarizability of fluorine leads to weak Van der Waals interactions, leading to very low boiling points compared to their homologous hydrocarbons [3, 4].

PFAS have had a profound impact on all aspects of the industry for their properties, mainly due to the stability of the C-F bond, characterized by a perfect overlap between the corresponding orbitals of carbon and fluorine, leading to a “self-stabilization” [3, 5]. Due to these properties, PFAS are used in different industrial fields (e.g., food packaging, waterproof textiles, cosmetics, anti-stick coatings for households, and cleaning products). However, this stability contributes to the high mobility and persistence of PFAS in the environment, bioaccumulating also in living organisms [1, 2]. Considering the great concern behind PFAS effects on human health [6–9], some of them were introduced in the Stockholm Convention as Persistent Organic Pollutants [10].

Their environmental behavior leads to a complex chain of transport in different matrices [11, 12]. Soil plays an important role as a reservoir for the transfer and accumulation of PFASs across various environmental compartments. Monitoring different PFAS classes within this matrix is essential, as it directly influences their bioavailability to plants and, consequently, their potential entry into the food chain [13, 14]. However, this task presents several challenges, since PFAS comprises thousands of compounds with diverse functional groups and physicochemical properties, contributing to analytical complexity. The heterogeneous nature of the soil matrix further complicates the extraction and analysis processes, requiring optimized sampling and extraction procedures to minimize potential losses or biases. Since PFAS are often present at trace levels, highly sensitive analytical instrumentation is also required to ensure accurate detection and quantification [15, 16].

Due to increasing regulatory restrictions on long-chain PFAS, recent research and industrial focus have shifted toward (semi-)volatile PFAS, which were initially regarded as safer alternatives owing to their lower bioaccumulation potential, reduced toxicity, and greater water solubility. However, these properties contribute to increased environmental mobility, facilitating their transport across various environmental matrices and raising new concerns. Recent studies have demonstrated that short-chain and (semi-)volatile PFAS can interfere with endocrine function and exhibit significant environmental persistence, making them resistant to degradation [17].

The analysis of these compounds can benefit significantly from the coupling of dynamic headspace extraction (DHS) with thermal desorption (TD), which provides a sensitive and efficient alternative to conventional approaches such as liquid–liquid extraction and solid-phase extraction, minimizing variability associated with sample preparation while enhancing analyte recovery. Additionally, the minimal use of solvents in TD reduces environmental impact and aligns with the principles of green analytical chemistry [18–20].

Given the ubiquity of PFAS in both the environment and laboratory materials, analytical methods must be designed to minimize background contamination. While liquid chromatography has been the standard technique for PFAS determination, gas chromatography coupled with mass spectrometry (GC-MS) represents a rising complementary approach for the analysis of PFAS, and specifically the (semi-)volatile homologues [18, 19, 21]. Additionally, non-targeted analysis of environmental samples using comprehensive two-dimensional gas chromatography (GC×GC-MS) offers enhanced separation power, selectivity, and sensitivity [22, 23]. This approach enables the detection of a broader range of PFAS, including trace-level compounds, as well as other environmentally relevant contaminants such as pesticides, halogenated compounds, and polycyclic aromatic hydrocarbons [24].

In this context, the aim of this study is to (I) develop and validate a novel method for the extraction and analysis of target (semi-)volatile PFAS in soil samples and (II) demonstrate the non-targeted capabilities of the proposed analytical approach. This research integrates the extraction efficiency of DHS using sorption tubes with the high sensitivity and identification capabilities of a GC(×GC)-TOFMS system. After initial method development and optimization, the TD-GC-TOFMS methodology was validated for the quantification of nine PFAS, namely fluorotelomer alcohols (FTOHs), fluorotelomer acrylates (FTAc), and alkyl sulfonamides (FOSA and FOSE). In addition, the research demonstrates the capability of the TD-GC×GC-TOFMS methodology for non-targeted screening of environmentally relevant compounds in soils.

## Experimental section

### Chemicals, supplies, and samples

The analytical standards for the target analytes, namely 4:2 FTOH (1H,1H,2H,2H-Perfluorohexan-1-ol), 6:2 FTOH (1H,1H,2H,2H-Perfluorooctan-1-ol), 8:2 FTOH (1H,1H,2H,2H-Perfluorodecan-1-ol), 10:2 FTOH (1H,1H,2H,2H-Perfluorododecan-1-ol), 8:2 FTAc (1H,1H,2H,2H-Perfluorodecyl acrylate), N-MeFOSA (N-Methylperfluorooctanesulfonamide), N-EtFOSA (N-Ethylperfluorooctanesulfonamide), N-MeFOSE (N-Methylperfluorooctanesulfonamidoethanol), and N-EtFOSE (N-Ethylperfluorooctanesulfonamidoethanol) were sourced from LGC Limited (Teddington, Middlesex, UK). Ultrapure water was produced using a Milli-Q system from Millipore (Bedford, MA, USA), and the C_7_-C_30_ linear alkane mix was obtained from Supelco (Bellefonte, PA, USA).

Stock standard solutions of each analyte (1000 mg L⁻^1^) were prepared in MS grade methanol (Supelco) and stored at −20 °C in the dark. Working solutions were prepared by diluting the stock solutions in ultrapure water to various concentrations.

Thermal desorption tubes (TDT) containing Tenax-TA (TA) as a sorbent (Supelco) were used for the DHS extractions. The desorption tubes were conditioned after each extraction and analysis cycle using a Phoenix T220 (REDshift s.r.l., San Giorgio in Bosco, Padua, Italy) with a ramp rate of 30 °C min⁻^1^ to 320 °C, held for 180 min, under a nitrogen flow of 100 mL min⁻^1^. Tube blank analyses were periodically conducted to prevent carryover.

### Dynamic headspace extraction

To evaluate the influence of different auxiliary solvents on PFAS recovery, three different conditions were examined: soil with no solvent addition, soil with added pure water, and soil with a 1:1 solution of pure water and methanol. Nine airtight headspace glass vials (20 mL capacity) were prepared, with three replicates for each condition. Firstly, 2.00 g of soil were added into the vials and spiked with the PFAS standard solution to achieve a concentration of 10 ng g^−1^. The vials were left overnight to allow the compounds to absorb into the soil matrix. Before extraction, nine spiked vials were divided into three groups (*n* = 3 each). Five milliliters of pure water were added to the vials of the first group, and 5 mL of the 1:1 water to methanol solution were added to the vials of the second group, while no solvent was added to the vials of the third group. Each vial was conditioned for 15 min at 30 °C with magnetic stirring (~ 800 rpm) and then connected to the sampling apparatus consisting of a pre-conditioned tube and a vacuum pump, which maintained an 80 mL min^−1^ sampling flow rate, for a total extraction volume of 1 L (ca. 12.5 min sampling time). For this research, Tenax-TA (a porous polymer of 2,6-diphenyl-p-phenylene oxide) was used as the sorbent material for its superior performance on the selected analytes, as previously demonstrated [20]. The pump was calibrated and the flow monitored with a flow meter during each sampling. After the extraction step, each tube was desorbed into the GC-TOFMS system and analyzed. Tukey’s test at a 95% confidence level was performed to compare the mean yields of each PFAS in the three different extraction conditions; the same approach was also applied to the total PFAS yield.

Regarding the extraction volume, three different volumes were evaluated (1 L, 2 L, and 5 L). Similarly to the solvent assessment, nine vials (three replicates for each volume) were spiked at a concentration of 10 ppb and equilibrated overnight. After matrix homogenization, 5 mL of a 1:1 solution of pure water and methanol were added, and the samples were extracted.

The real soil sample was also extracted using the selected volume under the same conditions and analyzed by GC(×GC)-TOFMS. Tukey’s test at a 95% confidence level was performed to compare the results.

### Instrumental experimental conditions

Both GC-MS and GC×GC-MS analyses were carried out using an Agilent 8890 GC coupled to a Pegasus BT 4D time-of-flight mass spectrometer (LECO Corporation, Mönchengladbach, Germany). A PAL System automated tube handling device (CTC Analytics AG, Zwingen, Switzerland) was used to place the tubes inside an Optic-4 multimode injector automatically (GL Sciences B.V., Eindhoven, Netherlands), featuring an inlet Peltier cooler and a liner exchanger (GL Sciences B.V.). The GC system also included a cryogenic trap (GL Sciences B.V.) for the secondary trapping and release stage. Chromatographic separation was achieved using an Rxi-5ms column (30 m × 0.25 mm × 0.25 μm d_*f*_) for the first dimension and an Rxi-17SilMS column (2 m × 0.25 mm × 0.25 μm d_*f*_) for the second dimension (both from Restek Corporation, Bellefonte, PA, USA). Helium (99.9999% purity) was used as the carrier gas.

After sampling, the TDTs were removed from the sampling device, capped, and placed in the PAL autosampler. Once placed in the thermal desorption inlet, a 4-min solvent venting step was applied (split flow of 20 mL min^−1^ and a column flow of 0.5 mL min^−1^, at 35 °C) and followed by the thermal desorption step, increasing the temperature from 35 °C to 300 °C with a quick ramp of 20 °C s^−1^, maintaining a split flow of 20 mL min^−1^ and a column flow of 1.5 mL min^−1^. The cryogenic trap temperature was maintained at −20 °C throughout the venting period (4 min) and the thermal desorption phase (45 s) to facilitate analyte trapping. Its temperature was then quickly raised to 300 °C at a rate of 50 °C s^−1^ (held for 3 min), allowing for the secondary release of the analytes.

The initial temperature of the GC oven was set at 40 °C (held for 1 min), then increased to 175 °C at a rate of 5 °C min^−1^. After reaching 175 °C, a rapid ramp of 30 °C min^−1^ was performed to reach the final temperature of 300 °C (held for 1 min), resulting in a run time of 33.10 min. The secondary oven and modulator temperature offsets were respectively set at + 10 °C and + 25 °C compared to the main oven. Regarding the GC×GC analysis, the oven temperature was set at 40 °C (held 1 min), then increased at 5 °C min^-1^ to 300 °C (held for 1 min), and the modulation period was 3 s (hot jet: 0.8 s; cold jet: 0.7 s). ; in 1D-GC analysis, the modulator was simply turned off. The MS transfer line temperature was set at 280 °C, and the electron ionization ion source temperature was set at 250 °C, with an ionization energy of 70 eV. The TOF was operated across a mass range of 29 to 600 *m/z*, using an acquisition delay of 180 s at a frequency of 10 Hz and 150 Hz for one-dimensional and two-dimensional analysis, respectively. Data acquisition and processing were performed using ChromaTOF software version 5.56 (LECO Corporation).

The quantification ion for each analyte was selected based on the mass providing the best selectivity and detectability, with a tolerance of 500 mDa. The quantifier ions are reported in Table 1.

For peak detection in non-targeted analysis, a signal-to-noise (S/N) of 10 was set. Target analytes were identified using standards (*Validated ID*). Putative identifications were based on mass spectral similarity and chromatographic features compared with the NIST23 library. Two levels of reliability were applied for the tentative identification of environmentally relevant analytes (Figure S4): a more reliable ID level, *Putative ID*^*i*^, was based on a combination of spectral (i.e., MS similarity > 85%) and chromatographic information (*i.e.*, RI tolerance ± 20, position on the 2D plot), while a weaker level of identification (*Putative ID*^*ii*^) was based only on the match of the MS fragmentation with reference databases (MS similarity > 75%) and the two-dimensional structured elution. The retention indices were calculated using a C_7_ to C_30_ alkane series and analyzed under the same conditions as the samples.

Blank system (with no sample tube desorption) was periodically run to exclude any carryover.

### Calibration and method validation

A five-level calibration curve was built for each of the nine PFAS using the previously described optimized conditions: 2.00 g of soil were added in the headspace vial and spiked with the mix of the nine PFAS standards in order to reach different final concentrations (10, 20, 30, 40, and 50 ng g⁻^1^). Three replicates were prepared for each level and left overnight for equilibration; then, 5 mL of a 1:1 mixture of pure water and methanol were added before extraction. The solutions were extracted using 1L volume under agitation (approximately 800 rpm), and the tubes were analyzed by GC-TOFMS. To validate the method linearity, accuracy (calculated using 25 ng g⁻^1^ as the percent difference between the expected and measured concentrations), precision (expressed as the relative standard deviation (RSD%) at the lowest level of the calibration curve), limit of detection (LOD), and limit of quantitation (LOQ) were calculated.

Even though an internal standard is generally helpful to monitor method variability, none was used in this study. However, our recent study showed very reproducible results on IS response [20] using the same extraction conditions for volatile PFAS.

## Results and discussion

### Optimization and assessment of extraction parameters

A recent work on DHS extraction of the same target chemicals in water samples [20] demonstrated the effectiveness of Tenax-TA in extracting the target PFAS; hence, the same trap material was maintained in the current research.

#### Solvent addition

Initially, spiked neat soil was used for DHS extraction of the target analytes. However, the recovery was not satisfactory; thus, the use of co-solvents was considered for the evaluation of the extraction.

**Fig. 1:**
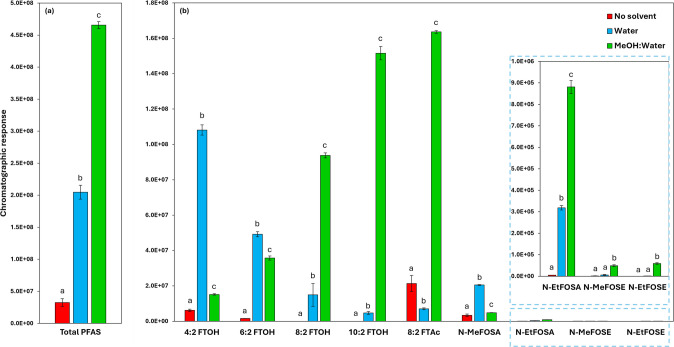
Evaluation of PFAS response from soil spiked at 10 ng g^−1^ (1 L extraction volume at 30 °C), using three different conditions (no solvent, pure water, and a solution 1:1 of pure water and methanol). **a** Total PFAS response (sum of EIC response of each target PFAS) and **b** individual target PFAS response (using the same quantifier ions reported in Table 1). Error bars represent the standard deviations, and Tukey’s test results are labeled as a, b, and c, where different letters denote statistically different means at a 95% confidence level

To assess the effect of solvent addition on PFAS recovery during DHS extraction, three experimental conditions were compared: no solvent addition (neat soil), addition of 5 mL water, as the solvent acts as an interface to remove the analyte from the matrix first [25], and addition of 5 mL of a 1:1 water:methanol mixture, similarly from the ASTM D7968-17a method, which employs this solvent system for liquid extraction of PFAS from soil [26]. The results obtained are presented in Fig. 1.

Tukey’s test was performed at a 95% confidence level to compare the mean yields of each PFAS across the different extraction conditions. The results indicated that dispersing soil particles in the water:methanol solution led to the highest overall PFAS recovery, with an overall 14-fold increase in chromatographic response compared to extraction without solvent (Fig. 1a). This condition also exhibited the lowest variability (1.6% RSD), whereas extraction without solvent resulted in the highest variability (18.1% RSD). Furthermore, the water:methanol solution provided the best results for most compounds, with the lowest variation, ensuring better reproducibility and reliability, although pure water provided better extraction efficiency for 4:2 FTOH, 6:2 FTOH, and N-MeFOSA. Examining the average response for the single FTOH homologues, a trend can be noticed: from the most volatile (4:2 FTOH) to the less volatile analyte (10:2 FTOH), the recovery increases using the methanol–water mixture, whereas a decrease in response is observed when pure water is used as a co-solvent (Fig. 1b).

#### DHS extraction volume

**Fig. 2:**
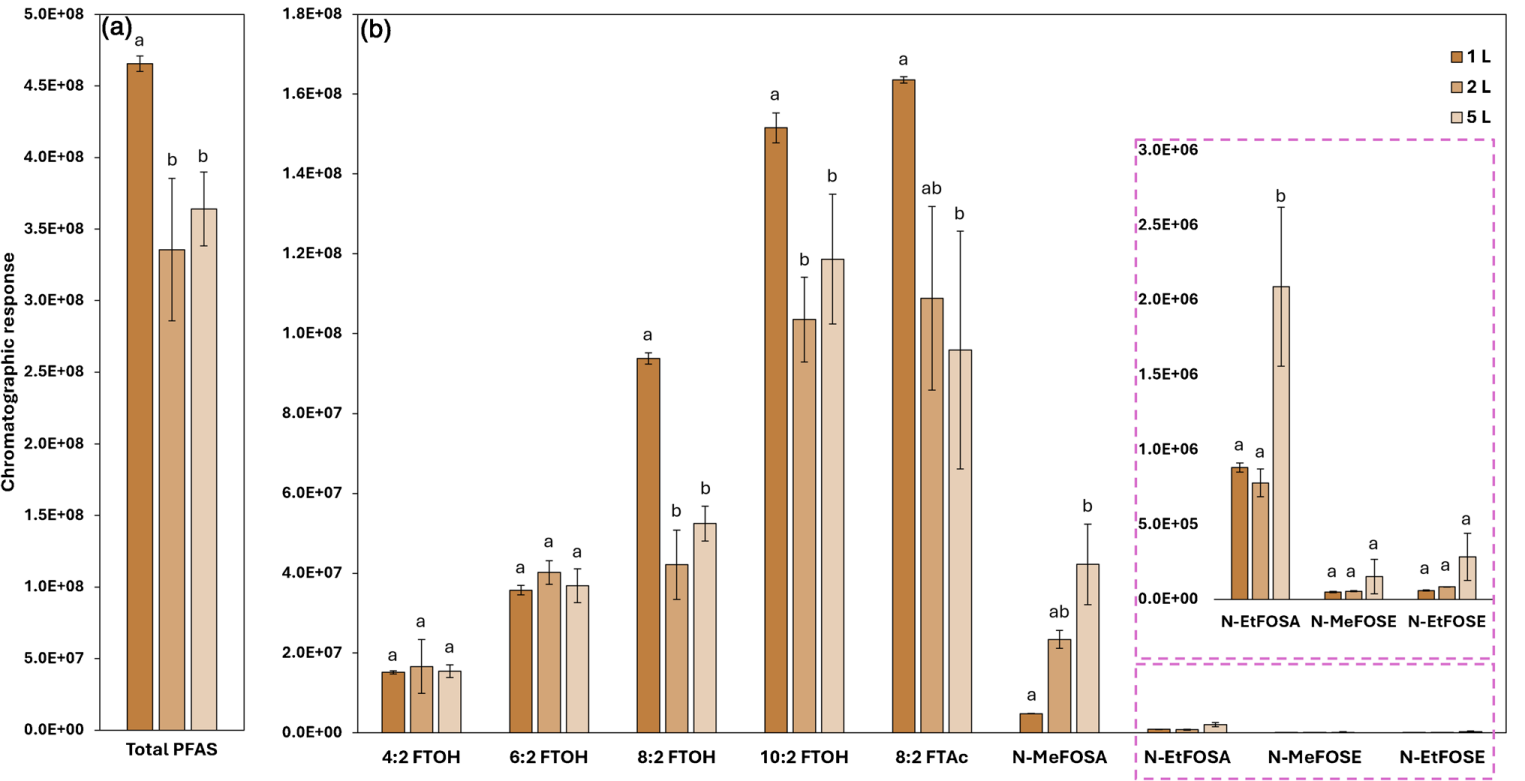
Evaluation of PFAS response from soil spiked at 10 ng g^−1^ and added with a 1:1 methanol:water solution, using different sampling volumes (1, 2, and 5 L). **a** Total PFAS response (sum) and **b** individual target PFAS response (using the same quantifier ions reported in Table 1). Error bars represent the standard deviations, and Tukey's test results are labeled as a, b, and c, where different letters denote statistically significant differences at a 95% confidence level

The headspace (HS) extraction volume was also evaluated. Specifically, 2.00 g of soil spiked with the nine target PFAS at 10 ng g^−1^ and mixed with a 1:1 methanol:water solution was subjected to extraction using volumes of 1 L, 2 L, and 5 L. The influence of the extraction volume on the recovery of target analytes is shown in Fig. 2.

Increasing the extraction volume resulted in higher variability, as indicated by a rise in relative standard deviation from 1.6% to 14.8%. Furthermore, the 1 L volume yielded the highest total PFAS recovery (Fig. 2a), with no significant difference observed between the 2 L and 5 L volumes. Individual PFAS responses (Fig. 2b) showed no significant differences across volumes for 4:2 FTOH, 6:2 FTOH, N-MeFOSE, and N-EtFOSE, although 1 L consistently showed the lowest standard deviations.

A trend was observed for the FTOH series, where recovery increased with volatility at the 1 L volume. For 8:2 FTOH, 10:2 FTOH, and 8:2 FTAc, lower volumes yielded higher and more consistent recoveries, suggesting displacement of lighter compounds at higher volumes, as reported by Borusiewicz et al. [27]. This pattern was further supported by FOSA and FOSE results, where 5 L extraction increased recovery but also increased variability, with N-MeFOSE showing the highest RSD (75.82%). Therefore, although higher volumes may enhance recovery for certain compounds, they are not reliable for consistent quantification of these compounds.

### Targeted analysis

A 5-point external calibration curve (ranging from 10 to 50 ng g⁻^1^) was constructed and validated under the optimized extraction conditions (as described in Section. "Calibration and method validation") in GC-TOFMS. Based on the previous results, the final DHS optimized method involved spiking 2.00 g soil samples, allowing them to equilibrate overnight, adding 5 mL of a 1:1 water-methanol mixture, and extracting with 1 L volume under ~ 800 rpm agitation.

The results for the validation parameters are summarized in Table 1.

**Table 1:** Calibration and validation parameters for the quantification model of each PFAS using GC-TOFMS

Analyte	*t*_*R*_(min)	Quantifier ion (*m/z*)	Linearity range (ng g^−1^)	*R*^2^	LOD (pg g^−1^)	LOQ (pg g^−1^)	Trueness^a^(%)	Precision^b^(%RSD)
4:2 FTOH	3.44	95	10–50	0.9973	35.96	108.97	−0.41	2.6
6:2 FTOH	5.24	95	10–50	0.9919	17.78	53.89	−2.89	3.3
8:2 FTOH	7.81	95	10–50	0.9982	12.16	36.85	−2.3	1.5
10:2 FTOH	10.72	95	10–50	0.9980	7.02	21.27	−3.82	2.5
8:2 FTAc	12.56	77	10–50	0.9980	13.17	39.90	2.14	0.5
N-MeFOSA	15.34	94	10–50	0.9967	34.55	104.71	−3.28	0.9
N-EtFOSA	16.00	448	10–40	0.9903	62.03	187.97	−3.38	3.4
N-MeFOSE	21.24	169	10–50	0.9953	211.09	639.66	−1.8	3.7
N-EtFOSE	22.56	540	10–40	0.9954	469.19	1421.80	3.36	5.8

^a^25 ng g^−1^, *n* = 3

^b^10 ng g^−1^, *n* = 3

Differently from the previous study [20], the quantitative ions were modified to enhance selectivity. This is indeed a choice which can be driven by the matrix and its interferences. For the FTOHs, *m/z* 95 was selected here instead of *m/z* 31 to minimize solvent-related interference, as traces of methanol (co-solvent in the optimized method) affect the quantitative performance of the FTOH class. For N-MeFOSA, N-EtFOSA, N-MeFOSE, and N-EtFOSE, the quantitative ions were changed from *m/z* 131 to *m/z* 94, 448, 169, and 540, respectively, to minimize interference from other coeluting compounds in the soil matrix. However, such coelutions would be resolved with the GC×GC separation due to its enhanced separation power.

The method demonstrated good linearity across the tested concentration range, with correlation coefficients ranging from 0.9903 to 0.9982. The LOD ranged from 7.02 pg g^−1^ to 469.19 pg g^−1^, and the LOQ varied from 21.27 pg g^−1^ to 1421.80 pg g^−1^, for 10:2 FTOH and N-EtFOSE, respectively.

The procedure was validated by assessing precision and accuracy, represented by the RDS% and average error, respectively. The RSD values ranged from 0.5% (for 8:2 FTAc) to 5.8% (for N-EtFOSE), confirming that this method provided consistent and reliable results for the PFAS classes under investigation.

### Non-targeted analysis

The non-targeted capability was assessed by desorbing the tubes under GC×GC-TOFMS conditions. Thanks to the broad selectivity of Tenax TA adsorbent and the separation power of the hyphenated and multidimensional technique, the resulting 2D chromatograms of real-world soil samples showed hundreds of analytes. This additional data was further explored, allowing for the suspect screening and a deeper insight into sample composition, particularly considering other contaminants of environmental concern.

In addition to the detection of PFAS in the initial target list (*Validated ID*), two supplementary identification levels were applied for the tentative identification:

- *Putative ID*^*i*^, assigned to compounds with spectral similarity > 85%, matching retention indices within ± 20 units of library values, and consistent positioning on the 2D chromatographic plane.
- *Putative ID*^*ii*^, assigned to compounds with spectral similarity > 75% and a consistent elution pattern in the 2D chromatographic space (see Electronic Supplementary Material Fig. S1).

**Fig. 3:**
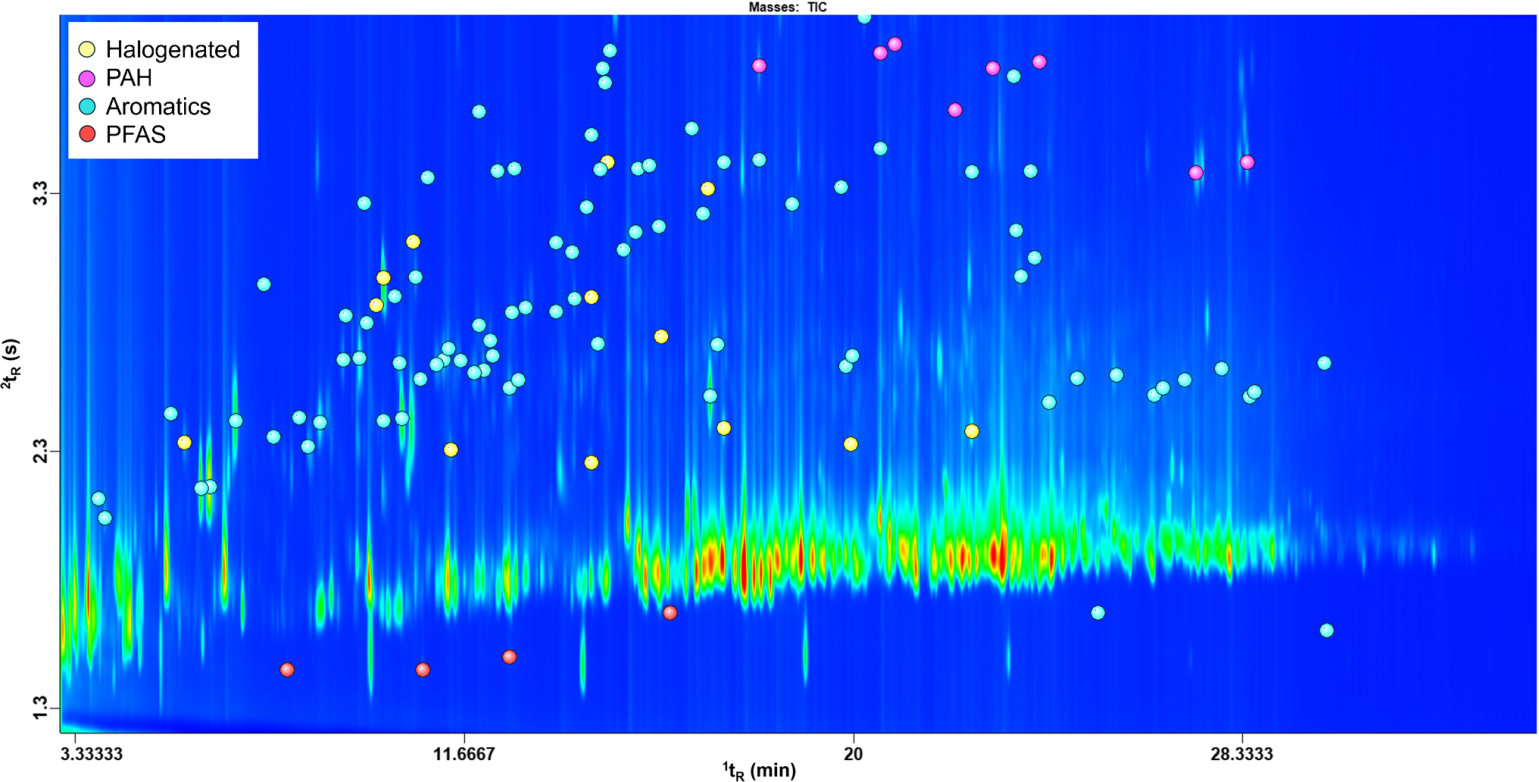
GC×GC-MS 2D plot of a soil sample. The color of the markers indicates the different classes of compounds tentatively identified under the *Putative ID*^*i*^ criterion*,* except for PFAS, which are the initial target analytes (*Validated ID*)

Figure 3 shows a 2D plot illustrating the identification of various environmentally significant compound classes. Traces of four target PFAS (8:2 FTOH, 10:2 FTOH, 8:2 FTAc, and N-MeFOSA) were confirmed (*Validated ID*) using analytical standards (red markers). The concentrations of 8:2 FTOH and 10:2 FTOH, quantified using the GC-TOFMS methodology described above, were 0.66 ppb and 0.97 ppb, respectively, while the other two compounds were detected below the LOQ. Additionally, 106 compounds of environmental relevance, classified as halogenated (yellow markers), aromatic (blue markers), and polyaromatic (pink markers), were identified based on the *Putative ID*^*i*^ criteria. A complete list of these tentatively identified compounds is provided in Electronic Supplementary Material Table S1.

**Fig. 4:**
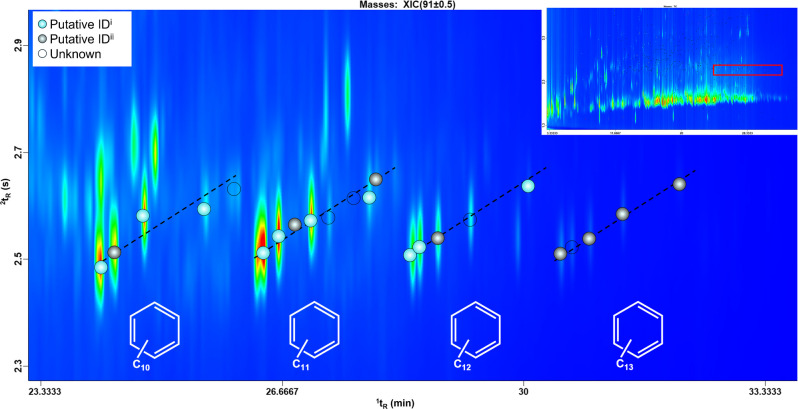
Expansion of an of homologous series of alkyl-benzenes from a representative GC×GC-MS plot. The blue and grey markers indicate those tentatively identified under the *Putative ID*^*i*^ and *Putative ID*^*ii*^ criteria*,* respectively, while the unfilled markers indicate the unknown compounds

The structured chromatographic elution reflects the analytes’ physicochemical properties, such as volatility (mostly driven by the 1D retention) and polarity/aromaticity (mostly driven by the 2D retention), facilitating compound classification and enhancing the identification process. As an example, a homologous series of alkyl-substituted benzenes (with 10 to 13 carbon atoms) elutes following a clear retention trend on the 2D plot, from the most to the least volatile compound (Fig. 4). This structured pattern strengthens confidence in tentative identifications and provides a more robust framework for recognizing structurally related compounds.

## Conclusion

This research represents an extension of our previous work for the development of sampling and analysis methods for (semi-)volatile PFAS in environmental samples [20]. Specifically here, a novel methodology to extract and analyze (semi-)volatile PFAS was developed, optimized, and validated on nine target compounds, including fluorotelomer alcohols (4:2, 6:2, 8:2, and 10:2 FTOHs), acrylates (8:2 FTAc), and alkyl sulfonamides (N-MeFOSA, N-EtFOSA, N-MeFOSE, and N-EtFOSE) in soil matrices. Through the DHS using thermal desorption tubes, the target analytes were extracted and then desorbed into a (multidimensional) gas chromatography-mass spectrometry system.

The proposed method provided suitable quantitative performance, reaching LOQ down to ppt levels (21.27–1421.80 pg g^−1^ range), with high precision (error 0.5–5.8 RSD%) in GC-TOFMS.

Despite the inherent complexity of the soil matrix, the combined power of multidimensional separation and time-of-flight mass spectrometry was effectively demonstrated through its application for non-targeted analysis. In a case study involving a real-world soil sample, the developed DHS-TD-GC×GC-TOFMS method enabled the detection of numerous additional compounds from environmentally significant chemical classes. These results highlight the method’s suitability not only for the targeted PFAS but also for broader non-targeted environmental screening applications.

Future studies will involve more (semi-)volatile PFAS classes and homologues, at the same time extending the range of sample matrices, in order to demonstrate the suitability of the DHS method for broader environmental monitoring.

## Supplementary Information

Below is the link to the electronic supplementary material.

Supplementary Material 1 (DOCX 73.0 KB)

## Data Availability

Data will be made available on request.
