# Supplementary material for: Development and optimization of PFAS extraction in soil’s headspace followed by multidimensional gas chromatography and mass spectrometry
Source: Anal Bioanal Chem. 2025 Dec 16;418(10):3137–45. doi: 10.1007/s00216-025-06266-4 (PMC13144168; doi:10.1007/s00216-025-06266-4)
Supplement: Supplementary file 1 — Supplementary Material 1 (DOCX 73.0 KB) [file 216_2025_6266_MOESM1_ESM.docx]

**SUPPLEMENTARY MATERIAL**

**Development and optimization of PFAS extraction in soil’s headspace followed by multidimensional gas chromatography and mass spectrometry**

Maria Chiara Corviseri^1^, Allan Dos Santos Polidoro^1^, Claudia Stevanin^1^, Luisa Pasti^1^, Flavio Antonio Franchina^*2^

^1^ Department of Environmental and Prevention Sciences, University of Ferrara, Via L. Borsari 46, 44121, Ferrara, Italy

^2^ Department of Chemical, Pharmaceutical, and Agricultural Sciences, University of Ferrara, Via L. Borsari 46, 44121 Ferrara, Italy

*Corresponding author: Flavio A. Franchina, PhD - Phone: +39 (0532) 455836 - E-mail address: [frnfvn@unife.it](mailto:frnfvn@unife.it), [flaviofranchina@gmail.com](mailto:flaviofranchina@gmail.com)


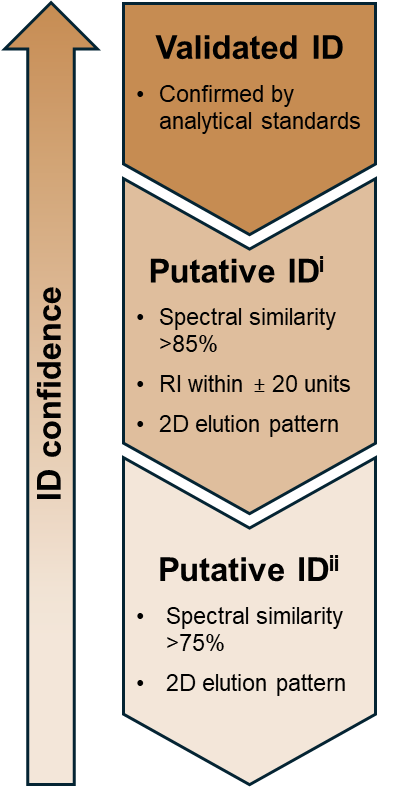


**Figure S1**. Schematics of the identification criteria used in the current study.

**Table S1.** Analytes from the GC×GC-TOFMS chromatogram in Figure 3 which were **validated** with reference standards (*Validated ID*) and those putatively identified combining a spectral similarity higher than 850‰, ^1^D retention index within ±20 units from reference values, and consistent positioning on the 2D plane (*Putative ID^i^* criterion). Class labels: (A) aromatic compounds; (H) halogen-containing compounds; (P) polycyclic aromatic compounds; (F) PFAS.

| **Compound** | **Class** | **Similarity**  **(‰)** | **Characteristic**  ***m/z*** | **^1^tR (min)** | **^2^tR (s)** | **RI_Exp_** | **RI_Lib_** |
| --- | --- | --- | --- | --- | --- | --- | --- |
| Pyrazine | A | 878 | 80.03 | 3.80 | 2.12 | 737.8 | 736 |
| Pyridine | A | 965 | 79.03 | 3.95 | 2.04 | 748.3 | 746 |
| 2-Picoline | A | 863 | 93.05 | 5.35 | 2.45 | 830.3 | 816 |
| Chlorobenzene | H | 969 | 112 | 5.65 | 2.34 | 844 | 849 |
| Ethylbenzene | A | 931 | 91.18 | 6.00 | 2.16 | 860 | 855 |
| *p*-Xylene | A | 945 | 91.14 | 6.20 | 2.17 | 869.2 | 865 |
| *o*-Xylene | A | 918 | 91.15 | 6.75 | 2.42 | 894.3 | 888 |
| Anisole | A | 852 | 108.05 | 7.35 | 2.95 | 917.6 | 921 |
| Cumene | A | 909 | 105.06 | 7.55 | 2.36 | 925 | 922 |
| **1H,1H,2H,2H-Perfluorodecan-1-ol** | F | 888 | 30.99 | 7.85 | 1.45 | 936.1 | 936 |
| Allylbenzene | A | 905 | 117.06 | 8.10 | 2.43 | 945.4 | 928 |
| Isocumene | A | 905 | 91.05 | 8.30 | 2.32 | 952.8 | 953 |
| *p*-Ethyltoluene | A | 901 | 105.06 | 8.55 | 2.41 | 962 | 954 |
| *o*-Ethyltoluene | A | 901 | 105.06 | 9.05 | 2.66 | 980.5 | 971 |
| *α*-Methylstyrene | A | 938 | 118.07 | 9.10 | 2.83 | 982.4 | 986 |
| Pseudocumene | A | 903 | 105.07 | 9.40 | 2.66 | 993.5 | 990 |
| Benzofuran | A | 964 | 118.04 | 9.50 | 3.26 | 997.2 | 1000 |
| *β*-Methylstyrene | A | 904 | 117.06 | 9.55 | 2.80 | 999 | 1011 |
| *m*-Dichlorobenzene | H | 932 | 145.96 | 9.75 | 2.87 | 1006 | 1018 |
| *sec*-Butylbenzene | A | 904 | 105.06 | 9.90 | 2.42 | 1011.2 | 1001 |
| *p*-Dichlorobenzene | H | 948 | 145.98 | 9.90 | 2.97 | 1011.2 | 1021 |
| *m*-Methylanisole | A | 911 | 122.07 | 10.15 | 2.90 | 1019.8 | 1020 |
| 1,2,3-Trimethylbenzene | A | 914 | 105.06 | 10.25 | 2.65 | 1023.3 | 1013 |
| *p*-Cymene | A | 926 | 119.17 | 10.30 | 2.43 | 1025 | 1025 |
| *o*-Dichlorobenzene | H | 948 | 145.97 | 10.55 | 3.11 | 1033.6 | 1043 |
| Indane | A | 943 | 117.06 | 10.60 | 2.98 | 1035.4 | 1029 |
| *m*-Cymene | A | 894 | 119.08 | 10.70 | 2.58 | 1038.8 | 1022 |
| **1H,1H,2H,2H-Perfluorododecan-1-ol** | F | 938 | 30.99 | 10.75 | 1.45 | 1040.5 | 1040 |
| Indene | A | 959 | 115.05 | 10.85 | 3.36 | 1044 | 1041 |
| *m*-Propyltoluene | A | 920 | 105.06 | 11.05 | 2.64 | 1050.9 | 1037 |
| 1-Phenylbutane | A | 895 | 91.05 | 11.20 | 2.66 | 1056.1 | 1054 |
| 1-Ethyl-3,5-dimethylbenzene | A | 917 | 119.08 | 11.30 | 2.70 | 1059.5 | 1059 |
| *n*-Octyl chloride | H | 880 | 91.02 | 11.35 | 2.31 | 1061.3 | 1059 |
| *o*-Propyltoluene | A | 917 | 105.06 | 11.55 | 2.65 | 1068.2 | 1051 |
| 1-Ethyl-2,3-dimethylbenzene | A | 911 | 119.08 | 11.85 | 2.61 | 1078.5 | 1090 |
| 2,4-Dimethylstyrene | A | 894 | 117.07 | 11.95 | 2.79 | 1082 | 1081 |
| *p*-Tolualdehyde | A | 909 | 91.05 | 11.95 | 3.62 | 1082 | 1079 |
| 1,2-Dimethyl-4-ethylbenzene | A | 915 | 119.08 | 12.05 | 2.62 | 1085.4 | 1085 |
| *p*-Cymenene | A | 922 | 117.06 | 12.20 | 2.73 | 1090.6 | 1090 |
| 1-Ethyl-2,4-dimethylbenzene | A | 862 | 119.08 | 12.25 | 2.67 | 1092.3 | 1075 |
| Methyl benzoate | A | 925 | 105.03 | 12.35 | 3.39 | 1095.8 | 1094 |
| **1H,1H,2H,2H-Perfluorodecyl acrylate** | F | 734 | 55.01 | 12.60 | 1.50 | 1104.5 | 1103 |
| 1-Methyl-4-(1-methylpropyl)-benzene | A | 865 | 119.08 | 12.60 | 2.55 | 1104.5 | 1096 |
| 1,3-Dimethyl-2-ethylbenzene | A | 901 | 119.08 | 12.65 | 2.84 | 1106.2 | 1087 |
| 2-Methylbenzofuran | A | 909 | 131.05 | 12.70 | 3.40 | 1107.9 | 1109 |
| 1-Methyl-4-(1-methylpropyl)-benzene | A | 895 | 119.08 | 12.80 | 2.58 | 1111.4 | 1096 |
| Durene | A | 919 | 119.08 | 12.95 | 2.86 | 1116.6 | 1115 |
| 1,2-Dimethylindan | A | 879 | 131.08 | 13.60 | 2.84 | 1139.2 | 1129 |
| 4-Methylindan | A | 893 | 117.07 | 13.60 | 3.11 | 1139.2 | 1141 |
| 5-Methylindan | A | 896 | 117.07 | 13.95 | 3.07 | 1151.3 | 1135 |
| Prehnitene | A | 913 | 119.08 | 14.00 | 2.89 | 1153.1 | 1143 |
| Tetralin | A | 914 | 104.06 | 14.25 | 3.25 | 1161.8 | 1155 |
| Nonyl chloride | H | 865 | 43.05 | 14.35 | 2.26 | 1165.2 | 1159 |
| Chloromesitylene | H | 922 | 119.08 | 14.35 | 2.90 | 1165.2 | 1157 |
| *α*-Acetoxytoluene | A | 873 | 91.05 | 14.35 | 3.53 | 1165.2 | 1164 |
| 1,4-Diethyl-2-methylbenzene | A | 920 | 133.1 | 14.50 | 2.72 | 1170.4 | 1164 |
| Ethyl benzoate | A | 891 | 105.03 | 14.55 | 3.40 | 1172.2 | 1172 |
| *o*-Acetyltoluene | A | 865 | 119.04 | 14.60 | 3.79 | 1173.9 | 1173 |
| 2,4-Dimethylbenzaldehyde | A | 877 | 133.06 | 14.65 | 3.73 | 1175.7 | 1182 |
| 1,2,4-Trichlorobenzene | H | 924 | 179.93 | 14.70 | 3.42 | 1177.4 | 1193 |
| Methyl *α*-toluate | A | 914 | 91.05 | 14.75 | 3.86 | 1179.1 | 1178 |
| 1-Methyl-4-(1-methyl-2-propenyl)benzene | A | 856 | 131.08 | 15.05 | 3.08 | 1189.5 | 1191 |
| 1-Methyl-4-(1-methyl-2-propenyl)benzene | A | 867 | 131.08 | 15.30 | 3.15 | 1198.2 | 1191 |
| Estragole | A | 926 | 148.08 | 15.35 | 3.40 | 1200 | 1196 |
| 4,7-Dimethylbenzofuran | A | 867 | 146.07 | 15.60 | 3.41 | 1209.1 | 1220 |
| *β*-Methyltetralin | A | 914 | 104.06 | 15.80 | 3.17 | 1216.3 | 1216 |
| Hexachlorobutadiene | H | 922 | 31.97 | 15.85 | 2.75 | 1218.1 | 1231 |
| **N-Ethylperfluorooctanesulfonamide** | F | 817 | 108 | 16.05 | 1.67 | 1225.4 | 1223 |
| *p*-Isopropylbenzaldehyde | A | 881 | 133.06 | 16.50 | 3.56 | 1241.8 | 1239 |
| 4,6-Dimethylindan | A | 861 | 131.08 | 16.75 | 3.22 | 1250.9 | 1232 |
| Bromomesitylene | H | 918 | 119.09 | 16.85 | 3.32 | 1254.5 | 1254 |
| *m*-Di-tert-butylbenzene | A | 915 | 175.23 | 16.90 | 2.52 | 1256.3 | 1249 |
| Hexylbenzene | A | 934 | 91.05 | 17.05 | 2.72 | 1261.8 | 1255 |
| Decyl chloride | H | 909 | 43.05 | 17.20 | 2.39 | 1267.2 | 1262 |
| 6-Methyltetralin | A | 893 | 131.08 | 17.20 | 3.42 | 1267.2 | 1261 |
| 5-Methyltetralin | A | 899 | 131.08 | 17.95 | 3.43 | 1294.5 | 1288 |
| *α*-Methylnaphthalene | P | 931 | 142.07 | 17.95 | 3.80 | 1294.5 | 1307 |
| Cyclohexylbenzene | A | 881 | 104.06 | 18.65 | 3.26 | 1321 | 1318 |
| 5-Ethyltetralin | A | 884 | 131.08 | 19.70 | 3.33 | 1361.2 | 1362 |
| Heptylbenzene | A | 927 | 92.05 | 19.80 | 2.63 | 1365.1 | 1366 |
| 1-Chloroundecane | H | 851 | 43.05 | 19.90 | 2.33 | 1368.9 | 1357 |
| 1-Methyl-2-n-hexylbenzene | A | 888 | 105.06 | 19.95 | 2.67 | 1370.8 | 1351 |
| Biphenyl | A | 966 | 154.07 | 20.20 | 3.99 | 1380.4 | 1381 |
| 5,7-Dimethyltetralin | A | 889 | 145.1 | 20.55 | 3.48 | 1393.8 | 1383 |
| *β*-Ethylnaphthalene | P | 852 | 141.07 | 20.55 | 3.85 | 1393.8 | 1391 |
| 1,7-Dimethylnaphthalene | P | 895 | 156.09 | 20.85 | 3.88 | 1405.6 | 1419 |
| *β*-Isopropylnaphthalene | P | 875 | 155.08 | 22.15 | 3.62 | 1458.2 | 1454 |
| *n*-Dodecyl chloride | H | 913 | 43.05 | 22.50 | 2.38 | 1472.4 | 1469 |
| 2,5,8-Trimethyltetralin | A | 886 | 159.11 | 22.50 | 3.39 | 1472.4 | 1471 |
| 1-Propylnaphthalene | P | 850 | 141.07 | 22.95 | 3.79 | 1490.6 | 1491 |
| *o*-Ethylbiphenyl | A | 850 | 182.11 | 23.40 | 3.76 | 1509.3 | 1494 |
| (R)-Cuparene | A | 889 | 132.09 | 23.45 | 3.16 | 1511.5 | 1505 |
| 2,4-di-t-Butylphenol | A | 882 | 191.14 | 23.55 | 2.98 | 1515.7 | 1514 |
| 1,1,4,5,6-Pentamethyl-2,3-dihydro-1H-indene | A | 874 | 173.13 | 23.75 | 3.39 | 1524.3 | 1522 |
| Calamenene | A | 872 | 159.11 | 23.85 | 3.05 | 1528.6 | 1523 |
| 2,3,6-Trimethylnaphthalene | P | 912 | 170.1 | 23.95 | 3.81 | 1532.8 | 1550 |
| (5-Decyl)benzene | A | 902 | 91.05 | 24.15 | 2.49 | 1541.4 | 1535 |
| (3-Decyl)benzene | A | 869 | 91.05 | 24.75 | 2.58 | 1567 | 1568 |
| Fluorene | A | 868 | 165.06 | 25.20 | 1.67 | 1586.2 | 1583 |
| (2-Decyl)benzene | A | 868 | 105.06 | 25.60 | 2.60 | 1603.5 | 1616 |
| (1-Butylheptyl)benzene | A | 902 | 91.05 | 26.40 | 2.52 | 1639.5 | 1632 |
| (1-Propyloctyl)benzene | A | 887 | 91.05 | 26.60 | 2.55 | 1648.5 | 1643 |
| 3-Phenylundecane | A | 871 | 91.05 | 27.05 | 2.58 | 1668.8 | 1670 |
| 1,3-di-iso-propylnaphthalene | P | 860 | 197.13 | 27.30 | 3.38 | 1680.1 | 1662 |
| (1-Methyldecyl)benzene | A | 944 | 105.06 | 27.85 | 2.62 | 1705.1 | 1708 |
| 2,6-Diisopropylnaphthalene | P | 871 | 197.13 | 28.40 | 3.42 | 1731.1 | 1728 |
| 6-Phenyldodecane | A | 904 | 91.05 | 28.45 | 2.51 | 1733.5 | 1726 |
| 5-Phenyldodecane | A | 897 | 91.05 | 28.55 | 2.53 | 1738.2 | 1730 |
| 2-Phenyldodecane | A | 883 | 105.06 | 30.05 | 2.64 | 1809.6 | 1808 |
| Ethylene glycol diphenyl ether | A | 913 | 77.03 | 30.10 | 1.61 | 1812.1 | 1811 |
